# Prediction of Cardiac Arrhythmias in Cancer Patients Treated with Immune Checkpoint Inhibitors Using Electrocardiogram

**DOI:** 10.3390/diagnostics15101235

**Published:** 2025-05-14

**Authors:** Alper Coskun, Ece Celebi Coskun, Ahmet Bilgehan Sahin, Fatih Levent, Eyup Coban, Fatih Koca, Seda Sali, Omer Furkan Demir, Adem Deligonul, Erhan Tenekecioglu, Erdem Cubukcu, Fahriye Vatansever Agca, Turkkan Evrensel

**Affiliations:** 1 Department of Medical Oncology, School of Medicine, Bursa Uludag University, 16059 Bursa, Turkey; alpercoskun@uludag.edu.tr (A.C.); absahin@uludag.edu.tr (A.B.S.); eyupcoban@uludag.edu.tr (E.C.); ademd@uludag.edu.tr (A.D.); erdemcubukcu@uludag.edu.tr (E.C.); evrensel@uludag.edu.tr (T.E.); 2 Department of Cardiology, University of Health Sciences, Bursa Yuksek Ihtisas Training and Research Hospital, 16310 Bursa, Turkey; fatihlevent85@hotmail.com (F.L.); drfatihkoca@gmail.com (F.K.); demiromerfurkan@gmail.com (O.F.D.); erhantenekecioglu@yahoo.com (E.T.); drfahriyevatansever@yahoo.com (F.V.A.); 3 Department of Medical Oncology, University of Health Sciences, Bursa City Hospital, 16250 Bursa, Turkey; sedasali@uludag.edu.tr

**Keywords:** arrhythmia, cancer, cardiotoxicity, electrocardiography, immunotherapy

## Abstract

**Background/Objectives**: Immune checkpoint inhibitor (ICI)-associated cardiovascular adverse events are relatively uncommon; they can be life-threatening, particularly when involving malignant ventricular arrhythmias. Electrocardiographic markers such as P-wave dispersion (PWD), QT dispersion (QTd), T-peak to T-end (Tp-e) interval, and Tp-e/QT and Tp-e/QTc ratios have been linked to an elevated risk of both atrial and ventricular arrhythmias and sudden cardiac death across various cardiac conditions. Monitoring these parameters may aid in identifying the risk of arrhythmogenic events in cancer patients undergoing ICI therapy. **Methods**: This retrospective cohort study analyzed 42 patients with cancer who received ICI therapy and had serial 12-lead electrocardiograms (ECGs) performed at baseline and at three-month intervals during the first year of treatment, from May 2022 to November 2023. ECG parameters including PWD, QTd, Tp-e interval, and Tp-e/QT and Tp-e/QTc ratios were measured and compared between baseline and follow-up time points. **Results**: The median follow-up duration was 5.3 months (range: 0.5–18.9 months). No statistically significant differences were observed in any of the ECG parameters between baseline and subsequent measurements (*p* > 0.05). One patient developed atrial fibrillation during the third month of treatment. Additionally, one patient exhibited a left anterior fascicular block, and another experienced frequent ventricular extrasystoles. No malignant ventricular arrhythmias were reported throughout the study period. **Conclusions**: This study found no significant changes in electrocardiographic markers associated with arrhythmia risk during ICI treatment. Larger, multicenter, prospective studies with extended follow-up are warranted to further elucidate the cardiovascular safety profile of ICIs.

## 1. Introduction

Immune checkpoint inhibitors (ICIs), a class of immunotherapeutic agents, have revolutionized cancer treatment by significantly enhancing response rates and prolonging survival across various malignancies, surpassing the efficacy of conventional chemotherapy regimens [1,2]. These agents have received regulatory approval for numerous cancer types, including melanoma, non-small-cell lung cancer (NSCLC), urothelial carcinoma, head and neck squamous cell carcinoma, and Merkel cell carcinoma [3]. By disrupting inhibitory immune signaling pathways—most notably cytotoxic T-lymphocyte-associated antigen 4 (CTLA-4), programmed cell death protein 1 (PD-1), and lymphocyte-activation gene 3 (LAG-3)—ICIs stimulate T-cell activation and potentiate antitumor immunity, a mechanism referred to as the inhibition of negative costimulatory signals [4,5,6,7]. Currently available ICIs include monoclonal antibodies targeting specific immune checkpoints: ipilimumab and tremelimumab inhibit CTLA-4; nivolumab, pembrolizumab, cemiplimab, and dostarlimab block PD-1; atezolizumab, durvalumab, and avelumab target PD-L1; and relatlimab acts against LAG-3 [5,6,7]. Despite their clinical success, ICIs are associated with immune-related adverse events (irAEs), which can affect a broad range of organ systems due to nonspecific immune activation. Unlike the toxicities associated with chemotherapy or targeted therapies, irAEs often resemble autoimmune or inflammatory disorders [8,9].

Cardiovascular irAEs, although less frequently reported, pose significant clinical challenges. In a cohort of 1813 ICI-treated patients, venous thromboembolism—comprising deep vein thrombosis and pulmonary embolism—was the most common cardiovascular complication, followed by myocardial infarction [10]. A meta-analysis encompassing 63 randomized clinical trials and over 32,000 patients highlighted increased incidences of myocarditis, pericardial disease, heart failure, dyslipidemia, myocardial infarction, and cerebrovascular ischemia in patients receiving ICIs [11]. Cardiac toxicities may present across a spectrum of clinical scenarios, from subclinical manifestations to rapidly progressing conditions such as fulminant myocarditis [12]. Reported cardiovascular presentations include myocarditis, pericarditis, arrhythmias, atherosclerosis, Takotsubo cardiomyopathy, and vasculitic processes [13,14].

Electrocardiography (ECG) remains an essential tool in evaluating arrhythmogenic risk. Parameters such as the PR interval, P-wave dispersion (PWD), QT dispersion (QTd), and the T-peak to T-end (Tp-e) interval offer insights into atrial and ventricular conduction abnormalities. The PR interval, denoting atrioventricular conduction time, though often benign in prolongation, has been associated with increased risks of atrial fibrillation (AF), pacemaker requirement, heart failure, and mortality [15,16]. PWD, defined as the difference between the longest and shortest P-wave durations across a 12-lead ECG, is a known risk marker for atrial tachyarrhythmias; a PWD ≥ 40 ms is considered an independent predictor of AF [17]. The QT interval, spanning from the onset of ventricular depolarization to the end of repolarization, reflects overall ventricular electrical activity. QTd, the inter-lead variation in QT duration, serves as an indicator of repolarization heterogeneity. While normal QTd typically ranges from 10 to 71 ms, values exceeding 100 ms are suggestive of pronounced electrical instability [18]. The Tp-e interval represents transmural repolarization heterogeneity, and its prolongation—along with elevated Tp-e/QT and Tp-e/QTc ratios—is associated with a heightened risk of ventricular arrhythmias and sudden cardiac death [19,20].

Among the various cardiotoxic effects of anticancer therapies, arrhythmias remain underrecognized. Anthracyclines have been linked to supraventricular arrhythmias and QTc prolongation irrespective of dosage, whereas cytotoxic agents like gemcitabine, cisplatin, and melphalan exhibit dose-dependent proarrhythmic potential, including QTc prolongation, torsades de pointes, and sudden cardiac death. Hormonal therapies such as selective estrogen receptor modulators and androgen deprivation agents may also contribute to QTc prolongation. Moreover, tyrosine kinase inhibitors have been implicated in early-phase torsadogenic effects and late-phase arrhythmias such as ventricular tachycardia and AF, sometimes without QTc prolongation [21]. Despite these associations, current evidence remains insufficient to establish a definitive causal link between specific anticancer agents and arrhythmias or to identify predictive markers for such events.

Given this background, the present study aims to assess arrhythmia risk in cancer patients undergoing ICI therapy through the serial evaluation of ECG-based parameters including PWD, QTd, Tp-e interval, and Tp-e/QT and Tp-e/QTc ratios.

## 2. Materials and Methods

This retrospective, observational study included adult patients (aged ≥18 years) who initiated ICI therapy for a cancer diagnosis and were followed at the Cardiology Department of Bursa Yuksek Ihtisas Training and Research Hospital and the Medical Oncology Department of Bursa Uludag University Faculty of Medicine between 1 May 2022 and 1 November 2023. Eligible patients had at least one baseline 12-lead ECG prior to starting ICI therapy and met the study’s inclusion criteria.

**Figure 1:**
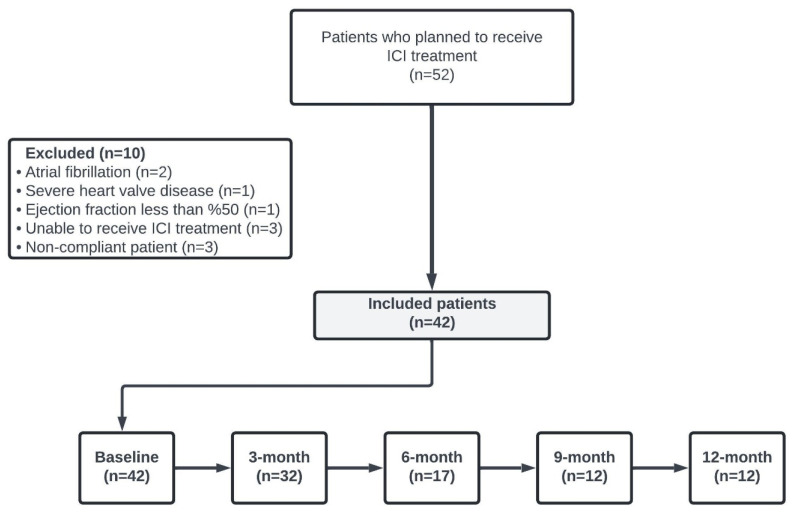
A flowchart of the study.

Exclusion criteria comprised recent (within the past 3 months) cardiovascular intervention for ischemic heart disease, advanced renal dysfunction (glomerular filtration rate < 30 mL/min), significant hepatic impairment, documented cardiomyopathy, severe valvular heart disease, left ventricular ejection fraction below 50% on transthoracic echocardiography, active sepsis, or a history of atrial flutter or atrial fibrillation. The patient selection process is illustrated in Figure 1.

Data on the demographic characteristics of the patients, details of ICI treatment regimen, the number of ICI cycles, follow-up periods, and survival status were extracted from hospital records and the electronic information system. Patients’ ECG measurements were assessed for baseline and subsequent 3-month intervals within the first year of treatment retrospectively.

Standard 12-lead ECG recordings were obtained using the GE Healthcare MAC 2000 device (General Electric Healthcare, Chicago, IL, USA) after a 15 min resting period. Recordings were performed at a paper speed of 25 mm/s and an amplitude of 10 mm/mV. PR interval, PWD, QTc, and QTd were measured across all 12 leads. The Tp–E interval, Tp–E/QT ratio, and Tp–E/QTc ratio were calculated from lead V5. All measurements were performed manually and independently by two experienced cardiologists who were blinded to patient data, and the maximum value was recorded for analysis. QTc was calculated using Bazett’s formula by dividing the QT interval by the square root of the R–R interval. Comparative analyses were conducted between baseline ECG parameters and those obtained during the treatment period. Rhythm abnormalities present at baseline or emerging during ICI therapy were retrospectively reviewed. Cardiac arrhythmias were classified according to the Common Terminology Criteria for Adverse Events (CTCAE), version 5.0.

## 3. Statistical Analysis

The statistical analysis of the study was carried out with IBM SPSS 25.0. Categorical variables were expressed as numbers and percentages, while continuous variables were stated with mean, standard deviation, median, minimum and maximum values. Kolmogorov–Smirnov and Shapiro–Wilk tests were used to determine normal distribution. The dependent sample t test was used as a double-repeat measurement test to compare variables that comply with normal distribution, the Wilcoxon test was used as a double-repeat measurement test to compare variables that do not comply with normal distribution, and the Mann–Whitney U test was used for comparative analyzes of subgroups. To evaluate the association between variables, the Chi-square test or Fisher exact test was applied. In the study, *p* < 0.05 was considered statistically significant.

## 4. Results

This study included 42 patients, with a median age of 63.5 years (27–82). Of these, 27 patients (64%) were male. The median follow-up was 5.36 months (0.46–18.86). The most common comorbid disease was hypertension, observed in 15 patients (36%). Hypertension was the most common comorbidity, affecting 15 patients (36%). The majority of patients (52%) received ICI therapy for NSCLC. Nivolumab was the most frequently used ICI agent (90%, *n* = 38). The median number of ICI cycles was seven [1,2,3,4,5,6,7,8,9,10,11,12,13,14,15,16,17,18,19,20,21,22,23,24,25,26,27,28,29,30,31]. The general characteristics of the patients are summarized in Table 1.

In the statistical analyses, no significant differences were found in ECG parameters when baseline values were compared with those measured at 3, 6, 9, and 12 months of treatment (*p* > 0.05). The results of the comparative analyses are shown in Table 3.

One patient developed sudden dyspnea and palpitation during the third month of ICI treatment. This patient, presenting to the emergency department, was diagnosed with new-onset AF and subsequently treated with electrical cardioversion, restoring sinus rhythm. Sinus rhythm was maintained throughout the follow-up period. Conversely, symptomatic sinus tachycardia was observed in 11 patients before treatment, with an additional 3 patients developing this condition during treatment. No cases of right bundle branch block were observed during follow-up. Left anterior fascicular block was observed in one patient during follow-up. This patient remained asymptomatic and no further cardiovascular events occurred during ICI treatment. After the 22nd cycle of nivolumab treatment, one patient developed palpitations and fatigue symptoms. Frequent ventricular extrasystoles (VESs) were observed on ECG. Twenty-four-hour rhythm Holter showed a total of 3200 VESs. Ventricular tachycardia, ventricular fibrillation and a pause longer than three seconds were not detected. Additionally, no patients developed first-degree, second-degree, or complete atrioventricular (AV) block or malignant ventricular arrhythmias either prior to or during treatment. Clinical characteristics and management of patients developed cardiac arrhythmias with treated ICIs are shown in Table 4.

A total of 15 patients (35.7%) received antihypertensive treatment. The most commonly prescribed agents were angiotensin receptor blockers, administered to six patients (14.3%). Additionally, eight patients (19%) were treated with combination antihypertensive therapy. Due to the small number of cases and the heterogeneity of regimens, a comparative analysis of antihypertensive medications was not performed. Five of our patients developed electrolyte imbalance at the beginning or during treatment, hyponatremia was observed in four patients, and hyperkalemia was detected in one patient. Statistical analysis could not be performed because the number of patients who developed electrolyte imbalance was small. When the use of anticancer drugs with known cardiotoxic side effects was evaluated, it was found that none of the patients had undergone treatment with anthracyclines, trastuzumab/pertuzumab, or anti-metabolites (5-fluorouracil or capecitabine) before ICI. Of the other cardiotoxic agents, 19 patients (45.2%) received microtubule inhibitors (taxanes and vinca alkaloids), while 8 patients (19%) received cisplatin. Information on the association between patient characteristics and cardiac arrhythmia can be found in Supplementary Table S1.

An analysis of pre-treatment ECG parameters revealed that patients with hypertension had significantly higher baseline PR interval values compared to those without hypertension (*p* = 0.034). Additionally, male patients had significantly higher baseline QT dispersion values compared to female patients (*p* = 0.007).

## 5. Discussion

ICIs have been associated with a spectrum of cardiovascular side effects, including arrhythmias. Although ECG changes are more commonly observed during ICI treatment, clinically significant arrhythmias, such as atrial and ventricular arrhythmias, are less frequently reported. The PWD, QTd, Tp-e interval, Tp-e/QT and Tp-e/QTc parameters are known to be independent risk factors for the development of cardiac arrhythmia. There are limited trials including these ECG parameters in cancer patients treated with ICIs. Therefore, our study was designed to contribute to the literature. The primary objective of our study was to evaluate the risk of arrhythmias in patients receiving ICIs by analyzing ECG parameters predictive of arrhythmogenic risk. In our study, no statistically significant differences were found in PWD, QTd, Tp-e interval, Tp-e/QT, or Tp-e/QTc parameters when pre-treatment measurements were compared to those during treatment (*p* > 0.05). No malignant ventricular arrhythmias were observed during the follow-up period.

Case reports in the literature have documented serious ICI-related arrhythmias, including complete and Mobitz type 2 AV block requiring permanent pacemaker implantation (22–24). In another case report, newly developed right bundle branch and left posterior fascicular block were observed in a patient receiving nivolumab for metastatic NSCLC. Although initially suspected to be secondary to myocarditis, the exact etiology remained unclear [25]. There is a limited number of clinical studies evaluating ECG parameters that predict the risk of cardiac arrhythmias in patients treated with ICIs (26–28). In a phase 2 study, no significant changes in pre- and post-treatment QTc values were observed in patients with solid tumors treated with different doses (0.3–10.0 mg/kg) of nivolumab (an anti PD-1 agent) [26]. In a study of 1818 patients with advanced solid tumors treated with avelumab (an anti PD-L1 agent), no statistically significant differences were observed when evaluating QTc and changes in QTc before and two weeks after avelumab infusion [27]. A study comparing ECG parameters at baseline and after 4–12 weeks in patients with stage III–IV malignant melanoma treated with either nivolumab monotherapy or combination therapy with nivolumab and ipilimumab (an anti-CTLA-4 agent) reported a significant increase in QTd in the combination therapy group. In both treatment groups, no changes were observed in heart rate, QRS duration, PR interval, or QTc interval during follow-up. A potential explanation for the significant QTd observed in the study by Pohl et al., which contrasts with our findings, may be that 51.2% of the patients in their cohort received combination therapy, whereas only one patient (2.4%) in our study was treated with this regimen. Notably, similar to our results, no significant change in QTc interval was observed in patients receiving anti-PD-1 monotherapy in the referenced study [28].

Although no study in the literature has been found that evaluates PWD in patients receiving ICI treatment, one study has reported significant increases in P wave duration and PWD in patients receiving 5-fluorouracil (5-FU), when the initial P wave duration and PWD measurements were compared with the 48 h measurements after bolus 5-FU and calcium folinate infusion. These changes were attributed to coronary ischemia and inter/intra-atrial conduction defects caused by the cytotoxic effects of 5-FU [29].

In a study involving 196 lung cancer patients treated with ICIs, it was reported that 23 patients (11%) experienced major adverse cardiovascular events (MACEs) and 7 of them (3.6%) developed supraventricular tachycardia [30]. In a retrospective study evaluating cardiovascular events in 230 patients with a median follow-up of 8 months, it was reported that one patient developed new-onset AF and one patient developed asymptomatic grade 2 type 1 AV block. These two patients were closely observed and monitored and treatment was not discontinued in either patient [31]. In another study evaluating the five-year follow-up of 268 patients receiving ICI therapy, AF was reported in four patients. This adverse event was observed more frequently in male patients and in those with comorbidities such as hypertension, diabetes mellitus, and hyperlipidemia. Moreover, among six patients with a prior history of paroxysmal AF, recurrence occurred within the first six months of treatment. These findings underscore the need for the careful monitoring of AF recurrence in patients receiving ICI therapy. Similarly to our study, all arrhythmic events in that cohort were clinically manageable, and no patient required treatment discontinuation. Additionally, as in our findings, no cases of arrhythmia were associated with concurrent myocarditis or suspected myocarditis [32]. In our study, one patient developed a grade 2 ventricular arrhythmia in the 11th month of ICI treatment and was followed up with close observation and follow-up without treatment interruption. Cardiotoxicity associated with checkpoint ICIs typically occurs early, but late cardiovascular events have also been reported [33,34,35]. In our study, a 72-year-old male patient with hypertension and diabetes mellitus was hospitalized with a newly diagnosed AF. The patient, who had preserved ejection fraction and no valvular pathology on transthoracic echocardiography, was observed to return to sinus rhythm with electrical cardioversion after transesophageal echocardiography and remained in sinus rhythm at follow-up.

The Tp-e interval, along with Tp-e/OT and Tp-e/QTc ratios, have been linked to a heightened risk of ventricular arrhythmias and mortality in conditions such as ST-elevation myocardial infarction, hypertrophic cardiomyopathy, and Brugada syndrome [36,37,38].

However, to our knowledge, no studies have evaluated these parameters in the context of ICI therapy.

This study has several important limitations that should be acknowledged. The most significant limitations are the relatively small sample size and the absence of a control group, which restrict the generalizability of our findings. Furthermore, transthoracic echocardiography and cardiac magnetic resonance imaging (MRI) data were not included in the analysis, limiting our ability to perform a comprehensive structural and functional cardiac assessment. Another important limitation is the unavailability of cardiac biomarker data, such as troponin I and N-terminal pro-B-type natriuretic peptide (NT-proBNP), which are clinically relevant for detecting subclinical myocardial injury. Due to the retrospective nature of the study, these biomarkers were not routinely measured; only two patients (4.8%) had troponin I levels recorded at baseline and at least once during treatment, and only one patient (2.4%) had corresponding NT-proBNP measurements. As a result, a meaningful statistical analysis regarding the association of these markers with arrhythmia risk could not be performed. In addition, the potential effect of ICI dosage on arrhythmia risk could not be evaluated. Among the 36 patients receiving nivolumab monotherapy, 29 were treated with a fixed dose of 240 mg every two weeks, while the remaining 7 received varying doses (ranging from 140 to 480 mg) and administration intervals (every two to four weeks). This heterogeneity in dosing regimens precluded a valid dose–response analysis. Moreover, the small number of patients in subgroups receiving other ICI regimens limited our ability to assess the impact of ICI type or treatment modality (monotherapy versus combination therapy) on arrhythmia development. Specifically, only two patients received pembrolizumab monotherapy; one received a combination of nivolumab and ipilimumab; one received ipilimumab monotherapy; one received a combination of nivolumab and cabozantinib; and one received nivolumab in combination with chemotherapy. Given the limited representation in these subgroups, comparative analyses were not feasible.

## 6. Conclusions

irAEs remain a significant challenge in contemporary oncology practice, with cardiac toxicities representing a particularly critical concern due to their potential for substantial morbidity and mortality. In this study, we investigated the utility of ECG parameters in predicting ICI-related arrhythmias. Our findings revealed no significant alterations in ECG markers indicative of an increased risk for atrial or ventricular arrhythmias during ICI therapy. The observed incidence of AF and VES was consistent with previously reported data; however, the absence of malignant arrhythmias in our cohort does not preclude the arrhythmogenic potential of ICIs. This study is subject to several limitations, including its retrospective design, small sample size, and short follow-up duration, all of which limit the ability to detect rare or delayed cardiac events. Furthermore, the lack of standardized cardiac monitoring protocols, such as serial ECGs, cardiac biomarkers, and advanced imaging, impairs a comprehensive assessment of subclinical cardiotoxicity. To validate these findings and to gain a more accurate understanding of the arrhythmogenic risks associated with ICI therapy, larger, multicenter, prospective studies with extended follow-up periods and standardized cardiac surveillance protocols are urgently needed.

## Figures and Tables

**Table 1:** The general characteristics of the patients.

Characteristics (*n* = 42)	*n* (%)
Age, Median (Minimum–Maximum)	63.5 (27–82)
Gender	
- Female	15 (35.7%)
- Male	27 (64.3%)
Comorbidity	
- Hypertension	15 (35.7%)
- Diabetes Mellitus	3 (7.1%)
- Ischemic Heart Disease	9 (21.4%)
Smoking Status	28 (66.7%)
Smoking (Package/Year), Mean ± SD	38.3 ± 19.6
Pathological Type	
- Non-Small-Cell Lung Cancer	22 (52.4%)
- Malignant Melanoma	14 (33.3%)
- RCC	2 (4.8%)
- Bladder	2 (4.8%)
- Skin SCC	1 (2.4%)
- Esophagus	1 (2.4%)
Type of ICI	
- Nivolumab	36 (85.7%)
- Pembrolizumab	2 (4.8%)
- Ipilimumab	1 (2.4%)
- Nivolumab + Ipilimumab	1 (2.4%)
- Nivolumab + Cabozantinib	1 (2.4%)
- Nivolumab + Carboplatin + Pemetrexed	1 (2.4%)
ICI Treatment Setting	
- Metastatic	37 (88.1%)
- Adjuvant	4 (9.6%)
- Neoadjuvant	1 (2.4%)
ICI Cycles, Median (Minimum–Maximum)	7 (1–31)

ICI, immune checkpoint inhibitor. ECG parameters, including PR interval, PWD, QTc, QTd, Tp-e interval, Tp-e/QT ratio, and Tp-e/QTc ratio, were evaluated at baseline (*n* = 42) and at subsequent intervals: 3rd month (*n* = 32), 6th month (*n* = 17), 9th month (*n* = 12), and 12th month (*n* = 12). Detailed ECG parameter values before and during treatment are presented in Table 2.

**Table 2:** The electrocardiography (ECG) parameter values of patients before and during immune checkpoint inhibitor treatment.

	Baseline (*n* = 42)	3rd Month (*n* = 32)	6th Month (*n* = 17)	9th Month (*n* = 12)	12th Month (*n* = 12)
PR (ms) (mean ± SD)	147.1 ± 16.4	147.7 ± 16.4	149.6 ± 14.9	151.0 ± 16.3	153.4 ± 17.2
P dispersion (ms) (mean ± SD)	39.5 ± 17.8	37.3 ± 13.6	37.0 ± 13.9	32.8 ± 7.7	36.3 ± 8.7
QT (ms) (mean ± SD)	428.4 ± 23.1	423.8 ± 17.1	427.7 ± 15.6	428.1 ± 16.4	429.3 ± 17.4
QT dispersion (ms) (mean ± SD)	41.0 ± 14.6	35.9 ± 9.8	37.1 ± 8.5	38.3 ± 5.8	40.8 ± 13.8
Tp-E interval (ms) (mean ± SD)	70.2 ± 18.4	70.0 ± 13.9	72.9 ± 16.9	73.3 ± 17.8	74.2 ± 18.3
Tp-E/QT (%) (mean ± SD)	0.19 ± 0.04	0.19 ± 0.05	0.18 ± 0.05	0.18 ± 0.05	0.19 ± 0.06
Tp-E/QTc (%) (mean ± SD)	0.17 ± 0.06	0.16 ± 0.04	0.16 ± 0.04	0.16 ± 0.04	0.17 ± 0.04

**Table 3:** Comparison of patients’ electrocardiography (ECG) parameter measurements before and during immune checkpoint inhibitor treatment.

	3-month PR	6-month PR	9-month PR	12-month PR	
Baseline PR	0.95 *	0.77 **	0.92 **	0.72 **	*p*
	3-month P Disp	6-month P Disp	9-month P Disp	12-month P Disp	
Baseline P Dispersion	0.54 *	0.62 **	0.83 **	0.28 **	*p*
	3-month QT	6-month QT	9-month QT	12-month QT	
Baseline QT	0.81 *	0.27 **	0.44 **	0.21 **	*p*
	3-month QTDisp	6-month QTDisp	9-month QTDisp	12-month QTDisp	
Baseline QT Dispersion	0.18 **	0.30 **	0.70 **	0.82 **	*p*
	3-month Tp-E	6-month Tp-E	9-month Tp-E	12-month Tp-E	
Baseline Tp-E	0.42 **	0.86 **	0.60 **	0.85 **	*p*
	3-month Tp-E/QT	6-month Tp-E/QT	9-month Tp-E/QT	12-month Tp-E/QT	
Baseline Tp-E/QT	0.28 *	0.26 **	0.28 **	0.93 **	*p*
	3-month Tp-E/QTc	6-month Tp-E/QTc	9-month Tp-E/QTc	12-month Tp-E/QTc	
Baseline Tp-E/QTc	0.10 **	0.44 **	0.24 **	0.81 **	*p*

* Paired samples *t*-test, ** Wilcoxon test.

**Table 4:** Characteristics, management and outcome of patients developed cardiac arrhythmias.

Patient Number	1	2	3
Age	68	82	51
Sex	Male	Female	Female
Primary Diagnosis	NSCLC adenocarcinoma	NSCLC adenocarcinoma	Cutaneous MM
Type of ICI Treatment	Nivolumab monotherapy	Nivolumab monotherapy	Nivolumab monotherapy
Number of ICI Cycles	8	22	6
Administration of ICI	240 mg every 14 days	180 mg every 14 days	240 mg every 14 days
Presentation	New-onset atrial fibrillation with rapid ventricular response diagnosed by ECG test	Palpitation, fatigue, frequent VES diagnosed by ECG test. 24 h rhythm Holter test showed a total of 3200 VES	Asymptomatic, left anterior fascicular block diagnosed by ECG test
Time to onset (days)	112	325	73
Grade	3	2	1
Management	Observed to return to sinus rhythm with ECV after TEE	Observing and closely monitoring and symptomatic therapy with β-B	Observing and closely monitoring
Outcome	Complete resolution	Partial resolution	Complete resolution
Follow-up	He remained in SR at follow-up and continued ICI therapy for another 11 cycles	After 1 month, 24 h rhythm Holter test showed total of 350 VESs. She continued β-B treatment at follow-up. She continued ICI therapy for another 9 cycles	She continued ICI therapy for another 7 cycles

β-B, beta blockers; ECG, electrocardiography; ECV, electrical cardioversion; ICI, immune checkpoint inhibitors; MM, malignant melanoma; NSCLC, non-small-cell lung carcinoma; SR, sinus rhythm; TEE, transesophageal echocardiogram; VES, ventricular extrasystole.

## Data Availability

The datasets used and/or analyzed during the current study are available from the corresponding author upon reasonable request.

## Supplementary Materials

The following supporting information can be downloaded at: https://www.mdpi.com/article/10.3390/diagnostics15101235/s1, Table S1: The association of patient characteristics with cardiac arrhythmia.
