# Supplementary material for: Prediction of Cardiac Arrhythmias in Cancer Patients Treated with Immune Checkpoint Inhibitors Using Electrocardiogram
Source: Diagnostics (Basel). 2025 May 14;15(10):1235. doi: 10.3390/diagnostics15101235 (PMC12109832; doi:10.3390/diagnostics15101235)
Supplement: Supplementary file 1 [file diagnostics-15-01235-s001.zip › ICI, ECG, SUPPLEMENTARY TABLE-1.pdf]

Supplementary Table 1: The association of patient characteristics with cardiac arrhythmia.

Characteristics	Arrhythmia		
	**Yes**<br>**N (%)**	**No**<br>**N (%)**	**p**
**Age**			
<65	1 (2.4%)	22 (52.4%)	0.581*
≥65	2 (4.8%)	17 (40.5%)	
**Sex**			
Female	2 (4.8%)	13 (31%)	0.287*
Male	1 (2.4%)	26 (61.9%)	
**Antihypertensive Medication**			
Yes	3 (7.1%)	12 (28.6%)	**0.040***
No	0 (0%)	27 (64.3%)	
**Diabetes Mellitus**			
Yes	0 (0%)	3 (7.1%)	1.000*
No	3 (7.1%)	36 (85.7%)	
**Hyperlipidemia**			
Yes	0 (0%)	4 (9.5%)	1.000*
No	3 (7.1%)	35 (83.3%)	
**IHD[1]**			
Yes	1 (2.4%)	8 (19%)	

No	2 (4.8%)	31(73.8%)	
**Smoking Status**			
Yes	1 (2.4%)	27 (64.3%)	0.254*
No	2 (4.8%)	12 (28.6%)	
**Treated with Cisplatin**			
Yes	0 (0%)	8 (19%)	1.000*
No	3 (7.1%)	31 (73.8%)	
**Treated with Anti Microtubul Agents**			
Yes	2 (4.8%)	17 (40.5%)	0.581*
No	1 (2.4%)	22 (52.4%)	

*Fisher's Exact Test; [1]Ischaemic Heart Disease.
